# Dengue Therapeutics, Chemoprophylaxis, and Allied Tools: State of the Art and Future Directions

**DOI:** 10.1371/journal.pntd.0003025

**Published:** 2014-08-28

**Authors:** James Whitehorn, Sophie Yacoub, Katherine L. Anders, Louis R. Macareo, M. Cristina Cassetti, Vinh Chau Nguyen Van, Pei-Yong Shi, Bridget Wills, Cameron P. Simmons

**Affiliations:** 1 Department of Clinical Research, London School of Hygiene and Tropical Medicine, London, United Kingdom; 2 Oxford University Clinical Research Unit, Hospital for Tropical Diseases, Ho Chi Minh City, Vietnam; 3 Department of Medicine, Imperial College, London, United Kingdom; 4 Armed Forces Research Institute of Medical Sciences U.S. Army Medical Component, Bangkok, Thailand; 5 National Institute of Allergy and Infectious Diseases, Bethesda, Maryland, United States of America; 6 Hospital for Tropical Diseases, Ho Chi Minh City, Vietnam; 7 Novartis Institute for Tropical Diseases, Singapore; 8 Centre for Tropical Medicine, Oxford University, Oxford, United Kingdom; 9 Dept. of Microbiology and Immunology, University of Melbourne, Parkville, Victoria, Australia; University of Rhode Island, United States of America

## Abstract

Dengue is the most common arboviral disease of humans. There is an unmet need for a therapeutic intervention that reduces the duration and severity of dengue symptoms and diminishes the likelihood of severe complications. To this end, there are active discovery efforts in industry and academia to develop interventions, with a focus on small molecule inhibitors of dengue virus replication that are suitable for therapy or chemoprophylaxis. Advancements in animal models of dengue virus infection together with the possibility of a dengue human infection model have further enhanced the platform for dengue drug discovery. Whilst drug discovery efforts gestate, there are ongoing clinical research designed to benefit today's patients, including trials of supportive care interventions, and descriptive studies that should improve the ability of clinicians to make an accurate diagnosis early in the illness course and to identify patients most at risk of progression to severe disease. This review provides a state of the art summary of dengue drug discovery, clinical trials, and supportive allied research and reflects discussions at the 2nd International Dengue Therapeutics Workshop held in Ho Chi Minh City, Vietnam, in December 2013.

## Introduction

The global dengue pandemic represents a major 21st-century public health challenge, with approximately 3,000,000,000 people living in areas at risk of transmission [1], [2]. Dengue causes individual suffering to those affected but also significant economic costs to endemic countries, as hospitals are frequently overwhelmed by dengue patients and those affected are unable to attend school or go to work [1].

Dengue is a systemic viral infection caused by any of the dengue viruses (DENV), of which there are four types, DENV-1–4. DENV are members of the Flaviviridae family and possess a single-stranded positive-sense RNA genome that encodes three structural proteins and seven nonstructural proteins. Infection of susceptible human hosts occurs after the bite of an infectious mosquito, usually *Aedes aegypti*. In susceptible human hosts, DENV replication likely occurs predominantly in cells of the reticuloendothelial system [3], [4]. Virus produced from infected tissues results in a viremia that can allow for onward transmission of DENV to naïve mosquitoes that take a blood meal [5]. Dengue is a self-limiting febrile illness, which, although often debilitating, typically resolves after 4–7 days of symptoms without major complications. Common symptoms include lethargy, headache, myalgia, nausea, vomiting, and rash. Viremia is apparent 1–2 days prior to the onset of symptoms, peaks during the first 1–2 days of fever, and then declines over the next 3–5 days until resolution.

A small proportion of people who develop dengue do experience severe complications during the illness. The most common complication identified is a significant increase in vascular permeability secondary to a transient vasculopathy; typically, this becomes apparent between the fourth and sixth day of illness, at a time when viremia is in steep decline or has resolved and the patient is afebrile [6]. In some patients, predominantly but not exclusively older children and young adults, significant vascular leakage occurs and can result in life-threatening hypovolaemic shock, with or without haemorrhage [1]. Various host and viral factors influence the eventual clinical phenotype of a DENV infection [7]. Current treatment is limited to fluid resuscitation and supportive care [8]. To prevent the complications that are typically observed between the fourth and sixth day of illness would require the administration of a therapeutic earlier in the course of illness. This raises a number of challenges—patients may not present to healthcare settings early in their illness, differentiating dengue from other febrile illnesses (OFI) is not easy, rapid diagnostic tests are relatively costly and not always available, and identifying patients at risk of severe disease and thus most likely to benefit from a therapeutic remains difficult. In addition, despite considerable research efforts, the pathogenesis of dengue remains incompletely understood [7]. These factors are challenges to the programmatic use of a dengue therapeutic in routine clinical practice.

To date, few trials of new specific therapeutic interventions have been conducted. However, this landscape is rapidly evolving, driven by a growing awareness of the scale of the disease burden [2], economic changes in endemic countries that are creating viable markets, and strong basic science that is supporting rational drug discovery and testing in small animal models. This article reflects discussions held at the 2nd International Dengue Therapeutics Workshop held in Ho Chi Minh City in December 2013 and attended by delegates from academia, industry, and funding agencies. It aims to illustrate the “state of the art” in dengue therapeutics discovery, clinical intervention trials, and enabling allied research.

### Target product profile for a dengue therapeutic

There remains an unmet need for an effective dengue therapeutic that can shorten the duration of illness, reduce the severity of common symptoms, and prevent the development of severe complications such as dengue shock syndrome (DSS). An example of a target product profile for an antiviral dengue drug is shown in Table 1. Desirable properties of a therapeutic candidate include low cost, ease of administration, and an excellent risk-benefit profile. While preliminary studies will typically be conducted in adults, it is important that children also be taken into consideration when developing the target product profile, as the disease burden of dengue falls most heavily on this age group.

**Table 1:** Target product profile for a dengue therapeutic.

Profile	Ideal
**Target population**	Adults and children (including infants and pregnant women)
**Dosing frequency**	Once daily
**Formulation**	Water-soluble tablet that can be dissolved in a small amount of liquid
**Pill burden**	One tablet daily
**Pharmacokinetic**	Half-life that enables once daily dosing
**Bioavailability**	Fast acting, high volume of distribution
**Interactions**	Minimal interactions with commonly used supportive care drugs
**Safety**	Well tolerated; no need for lab monitoring
**Stability**	No need for cold chain; 1–2-year shelf life at room temperature
**Cost**	To be investigated; needs to be affordable in dengue-endemic countries

## State of the Art in Small Molecule Therapeutic Trials in Dengue

Between 2007 and 2013, ten therapeutic trials in dengue patients were reported [9]–[18]. These have varied in size, quality, and design. Studies that have adopted a conventional randomised controlled approach have included trials of chloroquine, prednisolone, lovastatin, celgosivir, and balapiravir [16]–[20]. Chloroquine was investigated for both its antiviral properties and its potential ability to modulate the immune response to infection; however, the trial (n = 307 adult patients) did not demonstrate any clear antiviral or clinical benefits [16]. Prednisolone was investigated for its immunomodulatory properties with the hope that early initiation of therapy would prevent or attenuate severe manifestations of disease. The trial (n = 225 paediatric patients) was powered for safety but yielded no evidence of therapeutic benefit [18] and very limited evidence of attenuation of the host immune response [21]. Balapiravir, a prodrug of a nucleoside analogue, was clinically investigated as a candidate dengue antiviral on the basis of in vitro findings. However, the absence of a strong antiviral signal in 69 adult dengue patients led to cessation of the trial [17]. Subsequent work has indicated balapiravir is poorly metabolised to its active moiety in immune-activated cells, suggesting a possible explanation for the clinical trial outcome [22]. Another antiviral candidate, celgosivir, is a cellular glucosidase inhibitor. In vitro studies suggested celgosivir had antiviral activity against all four serotypes of DENV, and further in vivo work using a lethal mouse model showed celgosivir had antiviral activity against DENV-2 [23]. A clinical trial of celgosivir in 50 adult dengue cases suggested celgosivir was safe and well tolerated, but there was no evidence of an antiviral effect at the doses used [20], [24]. Inhibitors of HMG-CoA reductase, known as statins, were originally developed as lipid-lowering agents and have an established role in cardiovascular risk modification [25]. More recent research has shown that they have anti-inflammatory and endothelial-stabilising properties, and this has prompted the investigation of these drugs as adjunctive therapeutics for a range of conditions such as sepsis, pneumonia, and acute lung injury [26]–[28]. Based on the potential benefit of the properties of statins on the endothelium and as an immunomodulatory agent, it is plausible that they may have a beneficial effect in dengue. A trial of early lovastatin therapy in adult dengue cases is ongoing [19].

## State of the Art in Supportive Care Trials

Judicious fluid resuscitation is critical to the successful management of patients with severe dengue [8]. Isotonic crystalloid fluids (e.g., 0.9% normal saline and Ringer's lactate) are recommended for initial resuscitation of those with shock [1], [29], [30]. Colloid solutions (e.g., hydroxyethyl starch and Gelofusine) are suggested for patients with profound shock or for those who do not respond to initial resuscitation with crystalloids [8], [29]. There remain questions surrounding the optimal fluid management of the critically ill dengue patient, particularly for the estimated 30% of DSS cases who suffer recurrent episodes of shock [31], [32]. The safety of starch solutions, currently integral to resuscitation of severe dengue patients in many endemic countries, has also been called into question [33], [34]. While these safety concerns have generally arisen in elderly patients, often with comorbidities, and thus may not be relevant to the management of previously healthy children and young adults, they make the establishment of a clear evidence base for the optimal fluid resuscitation of patients with single or recurrent episodes of shock ever more pressing. In addition, the previous fluid intervention studies in dengue were all conducted in children; given the diverse epidemiology of dengue, with adults representing the majority of cases in some countries, it is important that future fluid trials include adult patients [35], [36].

Thrombocytopenia is almost universally observed in patients with dengue [1]. Despite a lack of evidence, prophylactic platelets are commonly administered in the belief that they can prevent haemorrhage [37]. This practice is both costly and potentially dangerous, as it constitutes administration of a blood product and a fluid challenge at the point of infection when fluid balance is critical [38]–[41]. A recently completed trial (n = 87 patients) suggested that administration of prophylactic platelets has no therapeutic benefit and is associated with an increased risk of harm—three patients who received platelets developed severe transfusion reactions [42]. The Adult Dengue Platelet Study (ADEPT) trial aims to further clarify the evidence for the use of prophylactic platelets in dengue (ClinicalTrials.gov identifier NCT01030211).

## State of the Art in Discovery of Antiviral Therapies

Encouragingly, a number of institutions, both academic and pharmaceutical, are actively engaged in dengue therapeutic discovery and development.

### Novartis Institute of Tropical Diseases (NITD)

NITD has made a concerted effort to search for dengue antiviral candidates. NITD has investigated host and virus protein targets using both a cell-based infection approach and a target-based rational approach [43]. Although several interesting inhibitors with various modes of action have been identified, some of which have demonstrated in vivo efficacy in mouse models, as yet none have advanced to the point of a clinical trial [44], [45]. However, the knowledge gained from these efforts has provided a better rationale for ongoing dengue drug discovery.

### Unither Virology

Unither Virology is attempting to develop a broad-spectrum, host-enzyme-targeted antiviral drug using an iminosugar platform. Iminosugars inhibit host alpha-glucosidases that are required for viral glycoprotein modifications. Inhibition of cellular glucosidase suppresses viral replication by disruption of productive folding pathways of the envelope glycoproteins prM (the intracellular glycosylated precursor of M [membrane protein]) and E (envelope protein). Encouraging results were seen with the leading candidate, UV-4, in a mouse model of DENV infection [46].

### Monoclonal antibodies for dengue

Therapeutic antibodies are also being explored to block dengue virus infection. Several potent monoclonal antibodies have been developed, including one that selectively neutralises DENV-1 [47]. Two challenges were perceived for the antibody approach: (1) it is likely that a panel of antibodies will be needed to inhibit all four serotypes of virus, and (2) the relatively high cost and requirement for parenteral administration may limit deployment in many endemic countries. It remains to be determined whether a single antibody that can potently neutralize all four serotypes can be developed.

### University of Leuven

Drug discovery at the University of Leuven has focused primarily on viral proteins using a cell-based screening approach and a wealth of experience gained from their research on hepatitis C and HIV therapeutics [48]. Currently, the lead dengue antiviral candidate is an inhibitor of nonstructural protein 4B (NS4B), which shows antiviral activity across all four serotypes and in a variety of cell lines.

### University of Marseilles

Work at the University of Marseilles is mainly focused on the screening of potential inhibitors of viral RNA-dependent RNA polymerase activity. A panel of polymerase inhibitors with both enzymatic and cellular activities has been identified. Efforts are ongoing to improve the potency of these inhibitors, as well as to identify their binding sites through biophysical and structural studies.

### University of Queensland

Investigators at the University of Queensland have been using cell electrical impedance measurements as a correlate of cell fusion mediated by enveloped viruses. This novel approach provides a high-throughput screening platform for the investigation of potential inhibitors of viral fusion. Besides flaviviruses, the approach could be applied to screen for fusion inhibitors of other enveloped viruses.

### Monash University

Researchers at Monash University are exploring the possibility of using inhibitors of nuclear transport as anti-DENV compounds [49]. In DENV infection, nonstructural protein 5 (NS5) localises in the nucleus through an interaction with importin-α1/β1 (IMPα1/β1). Intriguingly, the antiparasitic agent, ivermectin, has been shown to inhibit this interaction and reduce viral production [50]. Interestingly, ivermectin was also reported to inhibit flavivirus helicase activity [51]. Furthermore, the well-established safety profile of ivermectin demonstrated through its use in mass drug administration programmes makes this an attractive therapeutic candidate to investigate further.

## State of the Art in Research Tools Supporting Dengue Drug Development

### Funding resources for developing dengue therapeutics

Several government funding agencies and research charities around the world are currently supporting research on dengue, including the United States National Institutes of Health (NIH), the European Framework Programme, the Wellcome Trust (United Kingdom), and the Agency for Science, Technology, and Research (Singapore). The NIH, mostly through the National Institute of Allergy and Infectious Diseases (NIAID), is currently supporting several research projects to elucidate the basic biology of dengue virus and the mechanisms of disease development and to develop and evaluate therapeutics, diagnostics, and vaccines. A list of NIH-supported projects on dengue can be found on NIH Research Portfolio Online Reporting Tools (RePORT) (http://report.nih.gov/index.aspx). There are different funding mechanisms at the NIH to obtain support for dengue research. These include the following: (1) grant mechanisms, some of which focus on international research or product development partnerships (http://www.niaid.nih.gov/researchfunding/ann/pages/opps.aspx), and (2) preclinical and clinical research resources. These research resources include dengue reagents, bioinformatic databases, in vitro antiviral screening, evaluation of therapeutics in animal models, therapeutic preclinical development services, and clinical evaluation. Information about NIAID research services and contact information can be found at http://www.niaid.nih.gov/labsandresources/resources/Pages/default.aspx.

A list of additional funding sources can be found at http://www.niaid.nih.gov/researchfunding/ann/pages/found.aspx.

### Mouse models of DENV infection

Mice deficient in interferon-α/β and interferon-γ receptor (AG129) are susceptible to DENV infection and can experience a fulminant and fatal infection under certain experimental conditions using DENV2 strains [52], [53]. Ongoing work aims to develop mouse models of disseminated disease with other DENV serotypes and to replicate the severe disease that occurs in primary infections in some infants born to immune mothers [54]. As such, mouse models like the AG129 system provide a valuable mechanism to evaluate inhibitors of DENV infection and replication under in vivo conditions. One limitation of mouse models, however, is that they do not replicate the temporal sequence of the virological and common clinical events seen in humans with severe dengue, in particular the occurrence of hypovolemic shock relatively late in the illness course at a time when DENV infection of tissues has very nearly or already resolved. It may be that the vasculopathy seen in some DENV-infected patients is a phenomenon unique to humans. Thus, dengue mouse models may have limited utility in assessing interventions that target the cascade of host-mediated responses that are believed to partly underlie the syndrome of severe dengue in humans.

### Nonhuman primates and dengue

Nonhuman primates (NHPs), such as rhesus macaques (*Macaca mulatta*), are naturally susceptible to DENV infection and develop a viremia of similar duration to humans, yet they rarely manifest clinical signs or symptoms [55]. A trial of nonpegylated interferon in rhesus monkeys yielded a delay to peak viremia but with no change in the overall area under the curve. In another trial of recombinant pegylated interferon therapy versus placebo, a log decrease in viremia was observed over several days with a trend towards improved viral clearance, but the magnitude of the response was not deemed to be clinically useful [56]. NHPs have rarely been used in dengue drug development, perhaps because of the cost and the scarcity of laboratories capable of performing such studies. However, several NHP models have been developed that address different dengue manifestations [56]–[58]. These models could be utilized as part of a rational drug development plan, particularly for the advancement of novel drug entities, including direct-acting antiviral agents. In these scenarios, NHPs could provide the opportunity to conduct prophylactic and therapeutic trials in an animal model in which the virological and immunological features of disease are likely more “human-like” than in mice.

### Dengue human infection models (DHIM)

US Army researchers are developing a DHIM, whereby flavivirus-naïve adults are experimentally challenged with a DENV-1 virus strain that previously proved insufficiently attenuated to be used as a vaccine candidate for the purposes of vaccine development [59]. This is not a novel concept. Over 700 subjects have participated in such trials spanning from 1902 until the present time [60]. Rederivation of the challenge strain is in process, and an initial small-scale human trial to demonstrate the safety of the strain is currently being planned.

The administration of a dengue human infection will be conducted with all the current safeguards to protect human volunteers. The study would be done under a Food and Drug Administration (FDA) Investigational New Drug (IND) application, with independent review by both scientists and ethicists. The human volunteers would be informed of the study design, why the study was being done, and of the risks and the benefits of being in the trial. One challenge for the DHIM is that it should replicate the features of naturally acquired DENV infection, and early studies suggest this is possible [60]. A DHIM would also support the fast-track development of potential therapeutics, as it would allow for experimentally controlled trials of chemoprophylaxis and therapy and for detailed pharmacokinetic and basic research studies in preselected individuals.

### Physiological endpoints in therapeutic studies

As early phase research of novel therapeutic candidates is often exploratory and focused on safety, it is necessary to investigate surrogate markers of clinical impact rather than the major clinical complications themselves, e.g., DSS. Sensitively measuring the endothelial response to specific interventions in early-phase clinical trials would be ideal. Various noninvasive techniques have been developed that assess functional properties of the endothelium [61]. Peripheral artery tonometry is a user-independent method of measuring endothelium-dependent microvascular reactivity. Changes in microvascular reactivity have been shown to correlate with disease severity and outcome in sepsis and malaria [62], [63]. Alternatively, techniques such as videomicroscopy can be used to directly evaluate microcirculatory networks and perfusion status; studies using this method have demonstrated altered microcirculation in severe sepsis, again with correlations with the severity of organ dysfunction and outcome [64], [65]. These techniques have been used to monitor responses to particular therapies in other infectious diseases and may have a role as proxy endpoints for clinical trials in dengue [66]–[68]. It is possible that using noninvasive techniques that assess endothelial function and microcirculation may provide useful correlates of the capillary leak that is characteristic of severe dengue—ongoing observational research aims to evaluate this potential role [63], [69].

### Clinical descriptive studies and diagnostic/prognostic signs and symptoms

A number of important issues complicate the management of potential dengue cases in endemic areas. First, given the nonspecific nature of the symptoms and signs during the early febrile phase, establishing a firm clinical diagnosis without reliance on expensive diagnostics is difficult. Second, prediction of risk for the development of complications such as shock due to systemic vascular leakage is currently poor. A prospective multicentre observational study, aiming to enrol 10,000–12,000 outpatients presenting with a febrile illness consistent with possible dengue, is currently underway in seven countries across Southeast Asia and Latin America and is expected to report in 2016 (www.idams.eu). The availability of improved strategies for early diagnosis and risk prediction, ideally using a simple laboratory or a laboratory and clinical algorithm, would not only greatly facilitate patient triage but likely also enhance the productivity of clinical trials of early therapeutic interventions by focusing enrolment towards patients at greatest risk of complications. In high-risk patients, it may be easier to differentiate the treatment effect between the existing standard of care versus the standard of care plus the clinical intervention. From a practical perspective, a clinical trial enrolling patients at the highest risk of developing complications could reduce the size, duration, and cost of dengue randomised controlled trials, since the patient population would be enriched for the main endpoints of clinical relevance and interest.

## State of the Art in Prospects for Dengue Chemoprophylaxis

The concept of chemoprophylaxis for dengue has not been widely discussed as yet. Nonetheless, there are plausible scenarios and population groups that might benefit from an orally available chemoprophylactic agent. For example, aid workers, missionaries, and military travellers to dengue epidemic settings or “at risk” individuals living in areas of focal transmission in an endemic setting might benefit from chemoprophylaxis. Prophylactic delivery of a dengue antiviral compound has the theoretical advantage of interrupting the course of infection earlier than a therapeutic drug, i.e., prior to the development of peak viremia. However, a chemoprophylactic agent would need to be orally available, have an extremely good safety profile, and possess pharmacokinetic characteristics that allow for relatively infrequent dosing. Additional issues requiring further consideration, particularly for endemic settings, concern the identification of suitable target groups, the duration of dosing, and the risk-benefit ratio.

## Conclusions

There remains an unmet need for an effective dengue therapeutic or prophylactic, particularly in light of the continuing geographic expansion of dengue and the lack of an effective vaccine [2], [70]. This article has summarised the state of the art in the field of dengue therapeutics and the potential future landscape of therapeutic research. Increased collaboration between academia and industry, perhaps through public-private partnerships, is likely to be necessary for the successful development of an effective dengue therapeutic [43]. There remain many questions, foremost of which is whether an antiviral intervention can have a positive clinical impact when peak viremia typically occurs in the first 48 hours of illness and before most patients seek medical care. The dengue drug development field will benefit from a consensus on methodological approach in the conduct of early phase trials, common case report forms, and data sharing; efforts to provide these to the research community are underway [71]. In the long run, it is likely that a dengue therapeutic (or prophylactic) small molecule would fit into a “package” of dengue control measures comprising immunisation, vector control, and individual therapy.

**Box 1. Key Learning Points**

- There is an unmet need for specific interventions to improve the clinical management of dengue.
- Ongoing clinical research aims to improve diagnosis and prognosis and to test the efficacy of repurposed drugs.
- Discovery and development of specific therapeutics is directed at inhibiting virus replication or modifying host physiology.
- Therapeutic development is supported by animal models, and a human dengue virus challenge model is under development.

**Box 2. Top Five Papers in the Field**

- Thomas SJ (2013) Dengue human infection model: Re-establishing a tool for understanding dengue immunology and advancing vaccine development. Hum Vaccin Immunother 9: 1587–1590.
- Schul W, Yip A, Shi PY (2013) Testing antiviral compounds in a dengue mouse model. Methods Mol Biol 1030: 269–281.
- Simmons CP, Farrar JJ, Nguyen vV, Wills B (2012) Dengue. N Engl J Med 366: 1423–1432.
- Low J, Sung C, Wijaya L, Wei Y, Rathore A, et al. (2014) Efficacy and safety of celgosivir in patients with dengue fever (CELADEN): A phase 1b, randomised, double-blind, placebo-controlled, proof-of-concept trial. Lancet Infect Dis 14: 706–715.
- Bhatt S, Gething PW, Brady OJ, Messina JP, Farlow AW, et al. (2013) The global distribution and burden of dengue. Nature 496: 504–507.
